# Illu-Shoal Choice: An Exploration of Different Means for Enrichment of Captive Zebrafish

**DOI:** 10.3390/ani13162640

**Published:** 2023-08-16

**Authors:** Alberto Mair, Marco Dadda, Akiyoshi Kitaoka, Christian Agrillo

**Affiliations:** 1 Department of General Psychology, University of Padua, 35131 Padova, Italy; marco.dadda@unipd.it (M.D.); christian.agrillo@unipd.it (C.A.); 2 Department of Psychology, Ritsumeikan University, Osaka 567-8570, Japan; akitaoka@lt.ritsumei.ac.jp; 3 Padua Neuroscience Center, University of Padua, 35131 Padova, Italy

**Keywords:** zebrafish, enrichment, visual illusion, Ouchi–Spillmann illusion, shoal choice

## Abstract

**Simple Summary:**

The well-being of captive fish is often undervalued, and additional studies and new tools for enrichment need to be produced. Using 2D shapes of zebrafish filled with three different visual illusions in place of natural striped coloration, we tried to create a realistic dummy conspecific that is able to trigger social response, focusing on perceptual features such as overall shape, depth cues, and dynamic perceptions. When compared with a control stimulus that did not resemble a social companion, zebrafish showed no spontaneous attraction towards the images, either caused by a lack of perception of the illusion or by the fact that the illusory movement did not match the biological movement of real zebrafish. In future studies, we hope that we will manage to achieve this result using various illusory patterns. Creating realistic dummies of zebrafish could improve their well-being by acting as a source of social contact while excluding possible aggressive and stressful interactions. Moreover, it could be used as a reinforcement tool in training procedures.

**Abstract:**

Fish of any variety are nowadays being kept captive for several purposes, from recreational to alimentary to research. It is possible that we humans often underestimate or misunderstand the basic, natural needs of the species we use for our purposes. Sociality is likely to play an extensive and fundamental role in the quality of life of animals such as zebrafish. This study aimed to develop a dummy conspecific that included depth and motion illusions in order to assess whether these stimuli could represent a valid alternative to a conspecific in triggering shoaling behaviour in a well-known model in genetics and neuroscience, the zebrafish (*Dario rerio*). We thus replaced the natural livery of a zebrafish shape with three visual illusions: the Ouchi–Spillmann illusion, which generates an effect of local tilting motion; and another two which should create pictorial cues of tridimensionality. Via a binary shoal choice test, we assessed the time spent close to each of the three artificial dummies compared to neutral control stimuli such as grey ellipses. We found no preference for the illusory patterns, suggesting that the illusion was not perceived or, alternatively, that the perception of the illusion was not enough to elicit recognition of the dummy as conspecific and subsequent social behaviours.

## 1. Introduction

Over the past few decades, growing attention has been devoted to the life and welfare condition of captive animals, in both laboratories and natural parks. Several techniques have been employed to improve living conditions, meant to stimulate animals’ innate non-stereotypical healthy behaviours and improve their relationship with their environment [1]. Different sensory channels can be stimulated by scientists and keepers: vision, hearing, touch, and smell [2]. On several occasions, the enrichment is an object the animal can interact with and express instances of self-agency towards, ranging from spontaneous interaction to operant learning and proper training [3,4].

To avoid expensive and still-unresolved enrichment measures, in agreement with the 3R [5] principles that guide the use of animals in research, the biomedical and neuroscientific fields are shifting towards the use of animals whose needs are easier to fulfil because of their lower demands in terms of space, health, and enrichment. The zebrafish is a well-known model, and, over time, the number of zebrafish held captive in laboratories across the world has risen to several million in over 100 countries [6] due to the peculiar biological and morphological features that this animal model possesses. The relative ease of maintaining zebrafish populations and the apparent normal behavioural patterns displayed by the animals during their life can sometimes lead to neglect of important aspects of their lifestyle [7].

To refine our maintenance methods and improve the captive animals’ well-being, we should constantly advance our understanding of the animals’ needs. Several studies have been conducted to test natural preference for certain features of the artificial environment in which zebrafish are raised [8]. The results from different studies reported in this recent review are often discordant, indicating a possible lack of a comprehensive approach/paradigm to understanding zebrafish well-being. 

For example, zebrafish are widely considered a gregarious species because they naturally live in groups ranging from tens to hundreds of individuals [9]. Moreover, the onset of social contact is very precocious [10], at around 10 days post-fertilisation (dpf), shortly after the larvae become able to move and when the larvae’s perceptual system is still immature. Furthermore, isolation leads to a decreased immune response and increased stress levels in dangerous situations [11]. On the other hand, some studies have found that group living can raise stress levels [12], especially for certain types of individuals more prone to solitude [13]. Alternatively, group living could increase the level of stress via emotional contagion [14]. The probable causes span a range of concerns including overcrowding, opposite-sex conflicts for reproduction, and, more generally, dominance conflicts for resources such as sexual partners, food, and sheltering areas in the tank [15].

The company of conspecifics is certainly a natural, necessary feature of a healthy environment but presents a variability that could alter the enriching effects. In addition to the potential undesired effects of group living, some experimental procedures, such as training, require extensive periods of isolation.

These problems lead to the necessity of developing a neutral, social means of enrichment free from the potential negative effects of a real physical interaction. Artificial stimuli are another valid enrichment tool. An experiment by Saverino and Gerlai (2008) showed that video playback of moving conspecifics spontaneously attracts zebrafish, which prefer them to static images and to moving images of abstract figures. The relevance of these video dummies can be altered by manipulating aesthetic features of the stimulus. For instance, a strong elongation of the fish silhouette stimulates avoidance behaviours due to the increasing similarity to a possible predator shape. Conversely, yellow coloration, possibly associated with good health, increases the attraction to the conspecific dummy [16]. 

The main problem with video presentations is their practical implementation as an everyday tool to improve the well-being of captive zebrafish. Using a monitor for every tank that necessitates the treatment could be costly for laboratories.

Here we aimed to create an inexpensive 2D printable image of a zebrafish, capable of triggering a positive social response that can be used in laboratories regardless of their economic possibilities. In our preliminary investigation, simple, realistic images of zebrafish printed on paper failed to trigger any form of attraction; therefore, we decided to enhance the realism of the stimulus by modifying the internal livery. The visual system of zebrafish is in many ways similar to humans’, and on several occasions they have demonstrated susceptibility to visual illusions and other properties of the visual system comparable to mammals’ [17,18,19,20]. 

We chose different patterns and tried to mesh them into the livery in order to recreate the artificial features, such as motion and pictorial cues of depth, that we thought could have been relevant for zebrafish. We selected black-and-white striped patterns, similar to zebrafish skin, to create a stimulus that was as realistic as possible. We then presented them with non-realistic control stimuli and grey ellipses with the same dimensions and overall shape.

Our hypothesis was that dummies boosted with illusory features of movement and tridimensionality would be considered more attractive than the controls, resulting in more time spent in their proximity.

## 2. Materials and Methods

### 2.1. Subjects

We tested three mixed-sex groups of adult zebrafish numbering 56 in total: 16 zebrafish (approximately 12–16 months old) were tested in the first experiment (Ouchi–Spillmann illusion), and 20 in each of the other two experiments.

Fish were born and maintained at the comparative psychology laboratory (Animal Behavior and Cognition Lab) of the Department of General Psychology (University of Padova, Padova, Italy) in mixed-sex groups of 20 to 30 individuals (150 L in each tank). The aquariums were grey plastic tanks (70 cm × 45 cm × 55 cm) equipped with air filters, natural gravel, and live plants and maintained at a temperature of 25 ± 1 °C. The test tanks had no heating mechanism, as with all the tanks in our lab. The water temperature was maintained by ensuring a constant room temperature roughly 2 degrees above the desired water temperature.

The fish were fed twice daily: once with commercial food flakes and once with live brine shrimp (*Artemia salina*), on a 12:12 h light-to-dark (L:D) photoperiod under an 18-W fluorescent light. 

The local ethics committee of the University of Padova approved the study (permit number: 12/2021). 

### 2.2. Apparatus

**Figure 1:**
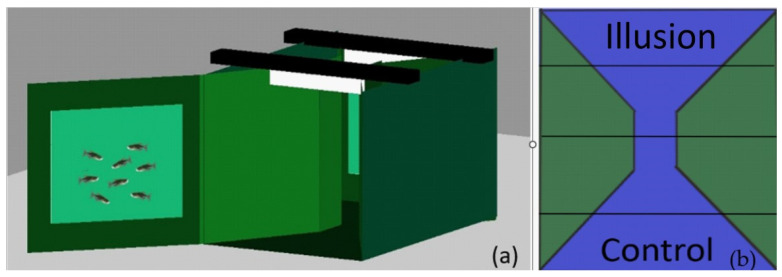
Visualisation of the apparatus (**a**) from the side (one of the two short sides is open for illustrative purposes) and (**b**) from above, depicting the hourglass shape.

The experimental tank, showed in Figure 1, was a 40 cm × 60 cm × 30 cm glass tank. The whole tank, except the two short sides, was covered with green opaque plastic to prevent the fish from seeing the outside. Moreover, two walls of green plastic were inserted into the tank. The narrow space between the two sectors was 10 cm wide. The reason for this, following previous shoal choice methods (16), was to partially prevent the simultaneous sight of the two stimuli. The fish’s choice of compartment had to imply a strong effect from the stimuli’s visual availability. The corners of the tank were rounded using the same green plastic to reduce the fish’s tendency to seek shelter when placed in a novel environment. All of these components came together to form an “hourglass” shape.

The experimental stimulus and the control stimulus were glued to two panels of the same green plastic and were attached at the two short sides of the tank during the experiment, alternating the presentation between the two positions.

Two 16-W LED lights (746 lm) were positioned on the top of the long sides of the tank to homogeneously illuminate the area. The recording camera was placed 50 cm above the water surface at the centre of the tank. 

### 2.3. Stimuli

The aim of the experiments was to create a dummy conspecific that is able to elicit a spontaneous positive social reaction in the experimental subject. To obtain this result, we tried to combine the artificial features that could be relevant for zebrafish into an overall semi-realistic dummy. All of the artificial stimuli selected had black-and-white striped patterns similar to zebrafish skin.

The three stimuli were produced using the vector graphic software programme Inkscape version 0.92.3 (https://www.inkscape.org, accessed on 15 June 2023). All three stimuli were created by inserting the artificial pattern into the body of the fish figure. The other features (head, fins, and size) were maintained.

**Figure 2:**
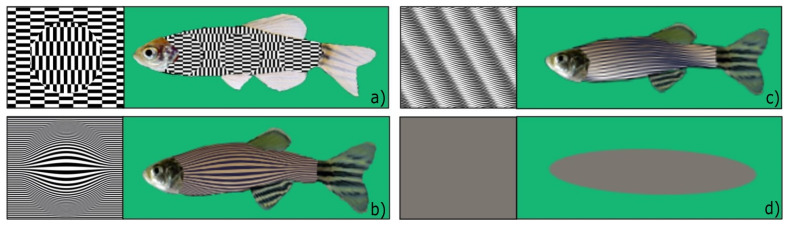
The four types of dummies used in our experiment: (**a**) Ouchi–Spillmann illusion, (**b**) depth illusion #1, (**c**) depth illusion #2, (**d**) grey ellipse as control.

Eight artificial fish of a particular illusory pattern were arranged in different orientations and inclinations on a rectangular sheet (30 cm × 21 cm) and subsequently glued to the panels, as shown in singular examples in Figure 2.

#### 2.3.1. Experiment 1: Ouchi–Spillmann Illusion [21] (Figure 2a)

This visual illusion consists of a disk made of horizontally oriented checks centred on a vertically oriented checked surrounding. The centre appears to slide relative to its surroundings depending on subtle eye and/or image movement. This motion illusion is quite robust in humans: it is enhanced by movement, it is also perceived monocularly, and, when blurred, it persists when presented near the periphery of the visual field and when the surroundings correspond only to the length of a check. We thought that these features could be promising for effectiveness in fish.

#### 2.3.2. Experiment 2: Depth Illusion #1 (Figure 2b)

We chose this black-and-white pattern to simulate the 3D effect of depth. The pictorial cue consists of the enlargement of the zebrafish body’s central stripes to render the impression of greater proximity. The movement of the stimuli produces a flickering that could contribute to drawing the subjects’ attention.

#### 2.3.3. Experiment 3: Depth Illusion #2 (Figure 2c) 

We chose this black-and-white pattern to simulate the 3D effect of depth. The pictorial cue consists of the non-linearity of zebrafish stripes. The different luminance could reflect the various light effects produced by a source of light impacting the body of a swimming zebrafish. The shades on the body produce an effect of greater proximity and tridimensional movement and produce a flickering that could contribute to drawing the subjects’ attention.

#### 2.3.4. Control Stimulus for Exp. 1–3 (Figure 2d)

The control was a grey ellipse (RGB: 123 119 120) of the same dimensions as a 4-cm-long zebrafish. The ellipse had no illusory movement, no body patterns to resemble a conspecific, and no distinctive fish features such as eyes, tail, and fins, considered of high importance in shape discrimination. Moreover, the control stimulus provided no depth cues, nor was it able to elicit any illusory motion in human observers.

### 2.4. Procedure

Experiments were performed during the daytime, from 10:00 (2 h after morning feeding) to 15:00 (2 h before evening feeding). Each subject was selected from the stock tanks and put into a small (1 L) plastic bucket for transportation to the experimental room.

After the time strictly necessary to move the fish from the maintenance to the experimental room (approximately 1 min), the subject was gently inserted into the experimental tank. After 60 s, the experiment started, and the time spent near the stimuli at the two short sides of the tank was taken as the dependent variable. We decided on a short habituation period to balance the subjects’ need for alleviation to netting and air exposition stress on one side and the necessity of avoiding excessive habituation that would have mitigated the motivation of this test, namely the need to shoal with conspecifics when placed in a new, empty, and thus slightly stressful environment. No subject showed signs of extreme stress, such as freezing, in their behaviour. 

When the experimental time elapsed (20 min), the subject was removed from the experimental tank using a fish net, transported through the plastic bucket back to the maintenance room, and placed into a new, post-experimental tank.

After removing the subject, 70% of the water in the experimental tank was replaced with water from three 150-L tanks enriched with gravel, plants, lamps, and a filter system to ensure the same quality/chemical composition of water and minimise possible interference from the previous subjects’ scent (e.g., hormones). Lastly, the positions of experimental and control stimuli were switched to prevent any possible bias originating from side preferences.

### 2.5. Video Analysis

The experiment was recorded via a camera placed above the experimental tank. Three trained observers analysed the videos offline. Each video consisted of preparatory routines (showing the ID of the subject and the position of stimuli and controls in the tank), 1 min of habituation, 20 min of experimental recording, and the final removal of the fish. 

The regions of interest were the two rectangles (10 cm × 40 cm each, following previous works [22,23] adjacent to the walls where the stimuli were hung, and the time spent in each sector was scored using the programme Ciclic Timer. The change of sector was determined by the passage of half of the subject’s body.

### 2.6. Data Analysis

Analyses were performed in R version 3.5.3 (The R Foundation for Statistical Computing, Vienna, Austria, http://www.r-project.org, accessed on 15 June 2023).

Preference for the target stimulus was evaluated by calculating the proportion of time spent by the subject in the choice area associated with the target stimulus, in detail:

Preference for the target stimulus = time near the target stimulus/(time near the target stimulus + time near the control stimulus).

In the same way, the preference for the lateral sectors was calculated and confronted with the central sector.

Student’s *t*-tests were performed to assess if the eventual preference for a sector was statistically significant against the null hypothesis of µ = 0.5.

Additionally, we performed the same *t*-tests with a Bayesian approach using the R package BEST to obtain a less dichotomous evaluation of the results.

The fluctuation of the preference across the 20 min of experiments was tested via a linear model with ID as the random factor and minutes as the predictor.

## 3. Results

Table 1 summarises the percentage of time spent in the three sectors by our experimental subjects. 

### 3.1. Do Fish Find the Stimuli Attractive? Lateral Sectors vs. Central Sector

In each experiment, the lateral sectors were preferred to the central one, especially considering that the area of the lateral sectors was only 40% of the total, the other 60% being the central sector.

**Figure 3:**
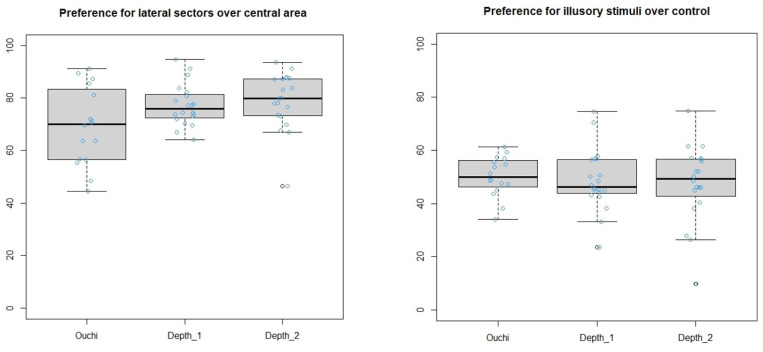
Boxplots representing the preference for lateral sectors over central area (**left**) and illusory stimuli over control (**right**).

The average percentage of time spent in the two lateral sectors compared to the central one was 69.18 ± 14.72 for the traditional version of the Ouchi–Spillmann illusion (t = 7.928, df = 15, *p*-value = 9.617 × 10^−7^, Cohen’s d = 1.98), 77.24 ± 7.88 for depth illusion #1 (t = 21.134, df = 19, *p*-value = 1.166 × 10^−14^, Cohen’s d= 4.73), and 78.78 ± 10.86 for depth illusion #2 (t = 16.024, df = 19, *p*-value = 1.713 × 10^−12^, Cohen’s d = 3.58). This assured us that the stimuli were explored by the fish for an extended period of time (Figure 3); thus, we had the opportunity to adequately compare them.

### 3.2. Do Fish Find the Illusory Pattern Attractive? Illusory Stimulus vs. Control Stimulus

No preference for any stimulus was found. The average percentage of time spent in the sector close to the target stimulus was 50.24 ± 7.58 for the Ouchi–Spillmann illusion (t = 0.1283, df = 15, *p*-value = 0.8996), Cohen’s d = 0.03), 48.78 ± 11.55 for depth illusion #1 (t = −0.4705, df = 19, *p*-value = 0.6433, Cohen’s d = −0.105), and 47.72 ± 14.38 for depth illusion #2 (t = −0.7085, df = 19, *p*-value = 0.4872, Cohen’s d = −0.158). 

**Figure 4:**
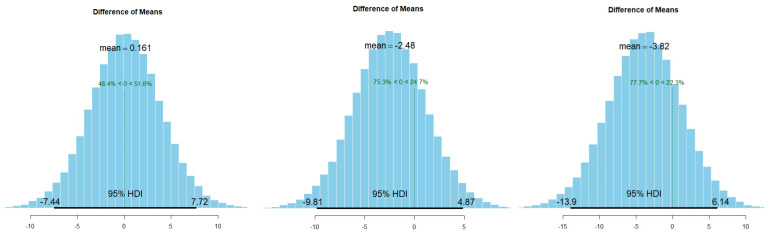
Posterior distributions of the MCMC simulations for Ouchi–Spillmann illusion (**left**), depth illusion #1 (**centre**), depth illusion #2 (**right**) preferences over the null hypothesis.

As mentioned in the method section, we tried to evaluate the weight of the slight differences in preference for the stimuli using Bayesian *t*-tests. Figure 4 depicts the distributions of the differences between our results and the null hypothesis after applying a Markov Chain Monte Carlo (MCMC) method, with 100,000 simulations.

The tests revealed no preference for the illusory stimulus in the Ouchi–Spillmann illusion experiment. 

The mean difference between the percentage of time spent in the region adjacent to the stimuli over the control is µ = 0.161. The probability that the illusory stimuli would be preferred to the control is 51.6%, very close to chance. In fact, the Bayes factor (BF) calculated to compare the likelihood of the presence of an effect against the null hypothesis is extremely low (BF = 0.25). Of comparably small size is the BF of depth illusion #1 (BF = 0.25) and depth illusion #2 (BF = 0.3), indicating evidence for absence of the stimuli’s effect. Nevertheless, the distributions of MCMC simulations of the two depth illusions result in negative differences of means between stimuli and control preference, indicating a tendency towards an effect that is opposite to the expected one. The difference of means for depth illusion #1 is µ = −2.28, resulting in a probability of preference for the control stimuli of 75.3%. The difference of means for depth illusion #2 is µ = −3.82, resulting in a probability of preference for the control stimuli of 77.7%.

**Figure 5:**
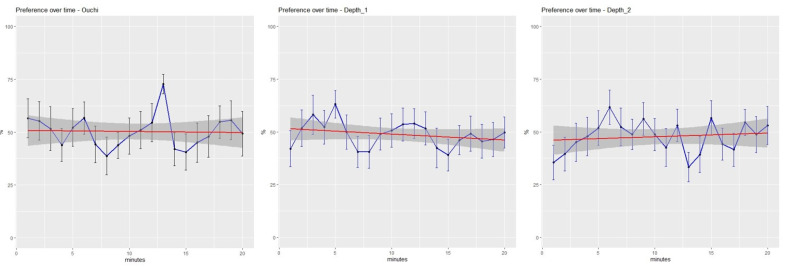
Percentage of time spent in the illusory sector close to Ouchi–Spillmann illusion (**left**), depth illusion #1 (**centre**), depth illusion #2 (**right**), over the 20 min of observation. Bars indicate S.D. The red lines and the dark grey shades indicate a smoothing function operated on the data obtained with the package ggplot2 (method = ”lm”) and their confidence intervals.

Finally, we assessed if fish preferences changed as a function of time. The linear models showed that fish preferences did not change across time (Ouchi–Spillmann: F = 0.023 Pr(>F) = 0. 879, depth illusion #1: F = 0.842 Pr(>F) = 0.359, depth illusion #2: F = 0.314 Pr(>F) = 0.575). As shown in Figure 5, the fluctuations of preference above and below the mean seem to be quite randomly distributed both within and between the experiments. 

## 4. Discussion

This study aimed to create 2D dummies of conspecifics that could be attractive to zebrafish. This would represent the first step to assess the validity of an efficient social, visual enrichment. In particular, we focused on two of the most important visual features of conspecifics: the presence of (illusory) movement and depth cues. To obtain these features on a 2D image, we employed black-and-white patterns to create a potentially realistic image of a conspecific that could attract the experimental subjects left in isolation. Our data analyses combined both *p*-value and Bayesian approaches and showed that our target stimuli were not more attractive than a control stimulus that did not include any feature of conspecifics. In the presence of null results, we can only speculate on the lack of difference in the proportion of time spent near target and control stimuli. First, fish could be poorly attracted by the target stimuli as these do not accurately represent real conspecifics. Previous studies have shown that visual stimuli with no odour or acoustic cues are effective as a proxy of conspecifics [16,24,25,26]. However, in these studies, body features (e.g., colour, texture, etc.) were similar to those of conspecifics. Here, in the attempt to add depth cues and illusory motion, we largely modified inner features of the dummy social companions, probably disrupting the perception of a real, healthy conspecifics. 

Other methodological issues may have played a role. The scoring technique only allowed us to count the time spent in each sector and calculate the preferences, possibly missing other relevant kinematic features such as a frame-by-frame position and a speed that could be captured by implementing efficient tracking technology. Depth and speed both have biological meaning [27] in zebrafish and could reflect dispositions that might be missed by analysing only the x and y positions of the subjects. Distance from the target stimulus is the most common dependent variable adopted in this field, but focusing only on this variable might have caused us to neglect other aspects of fish behaviour that could have indirectly revealed their preference for either stimulus. Another index that may unveil underlying processes is stress levels. In the mildly stressful situation of being alone in an empty tank with no enrichment, the (illusory) presence of conspecifics would mediate the production of stress-related hormones such as cortisol [11]. Measuring the quantity of cortisol in the water removed after each trial and matching it with the precise kinematic profile would give us a wider picture to interpret the results.

Alternatively, the illusions could fail to reproduce the desired effect of realism for several reasons. First, zebrafish perceive UV light and are naturally attracted to it [28]. A real conspecific could have a distinctive UV reflectance pattern when staying still and when swimming, which may help in the recognition of biological entities in an otherwise possibly turbid environment full of nonbiological distractors.

Secondly, the illusion presented could be based on some perceptual mechanism that zebrafish do not possess. In humans, the illusion requires checks of low spatial frequency; it persists with low luminance contrast and blurring of the inducing pattern but is absent at equiluminance [21]. The perception of the Ouchi–Spillmann illusion is thought to be independent from some magnocellular neuronal mechanisms yet to be identified that could be absent or reduced in the visual pathways of zebrafish.

Moreover, an important parameter for the strength of this illusion is the dimension of the circle compared to the dimension of the checks [29]. To obtain a visible central circle and inner sectors that could match the dimensions of zebrafish’s stripes, we had to expand it, limiting the space.

Another possible hypothesis for our experimental subjects’ lack of reaction to these stimuli is that these patterns produce effects localised inside the body of the conspecific. No effects produced a shift of the fish’s body shape relative to the background. Zebrafish and many other animals use immobility (e.g., freezing) to hide from predators. During freezing behaviour, a zebrafish continues to move its pectoral fins to maintain its position in the water. Thus, the global movement of the fish in the environment seems to be more relevant from the biological–evolutionary perspective than small local movements, even if constant and rapid.

Lastly, we did not assess the influences of personality trait scores with tests measuring relevant sources of individual variation [30]. Thus, we cannot rule out the possibility that different predispositions to sociality could have existed in our sample and affected the behavioural response and effectiveness of our enrichment stimuli. 

The route is long, and further investigation is required in this field. In the absence of any preference by fish for our target stimuli, we must be cautious in assuming that stimuli with an overall shape of conspecifics and depth cues and illusory motion in the inner part could be the powerful social enrichment we were hoping for.

## 5. Conclusions

The quest to refine animal welfare conditions is never ending and brings with it possible advancements in the comprehension of the perceptual mechanisms of the study’s animal subjects. Some natural preference could be hard-wired in the brain and senses of the animal and trigger a series of behaviours and conditions that enhance or diminish the animal’s well-being. 

In this study, we tried to capitalise on the natural predisposition of zebrafish to shoaling or at least the preference for group living. This is part of a set of studies aiming to trace the fundamental features of visual social recognition in zebrafish. We tried to simulate some realistic features such as movement and depth using some visual illusions, but the behavioural results we collected are largely inconclusive. We cannot firmly rule out the occurrence of methodological issues in stimuli presentation, but the most likely explanation is that the illusions are not perceived by the subjects, or if they are perceived, they fail to produce the effect of a realistic conspecific. 

Future studies should disentangle this issue and continue to advance the availability of information regarding zebrafish social and perceptual systems.

## Figures and Tables

**Table 1:** Mean percentages of time the spent in the different zones by the subjects.

	Ouchi-Spillmann Illusion	Depth Illusion #1	Depth Illusion #2
Illusion	34.36% ± 30.36	37.58% ± 31.44	37.87% ± 32.63
Center	30.82% ± 21.72	22.76% ± 16.26	21.06% ± 17.86
Control	34.53% ± 30.82	39.67% ± 31.76	41.85% ± 34.17

## Data Availability

The datasets are available upon request to the corresponding author.
